# Scanning the genome for gene single nucleotide polymorphisms involved in adaptive population differentiation in white spruce

**DOI:** 10.1111/j.1365-294X.2008.03840.x

**Published:** 2008-08

**Authors:** Marie-Claire Namroud, Jean Beaulieu, Nicolas Juge, Jérôme Laroche, Jean Bousquet

**Affiliations:** * Canada Research Chair in Forest and Environmental Genomics and Arborea, Forest Research Centre, Pavillon Charles-Eugène-Marchand, Université LavalQuébec, Québec, Canada G1K 7P4; † Natural Resources Canada, Canadian Forest Service, Canadian Wood Fibre Centre1055 du P.E.P.S., PO Box 10380, Stn. Sainte-Foy, Québec, Québec, Canada G1V 4C7; ‡ Centre for Bioinformatics and Computational Biology, Pavillon Charles-Eugène-Marchand, Université LavalQuébec, Québec, Canada G1K 7P4

**Keywords:** expressed genes, *F*_ST_, genome scan, local adaptation, SNP, white spruce

## Abstract

Conifers are characterized by a large genome size and a rapid decay of linkage disequilibrium, most often within gene limits. Genome scans based on noncoding markers are less likely to detect molecular adaptation linked to genes in these species. In this study, we assessed the effectiveness of a genome-wide single nucleotide polymorphism (SNP) scan focused on expressed genes in detecting local adaptation in a conifer species. Samples were collected from six natural populations of white spruce (*Picea glauca*) moderately differentiated for several quantitative characters. A total of 534 SNPs representing 345 expressed genes were analysed. Genes potentially under natural selection were identified by estimating the differentiation in SNP frequencies among populations (*F*_ST_) and identifying outliers, and by estimating local differentiation using a Bayesian approach. Both average expected heterozygosity and population differentiation estimates (*H*_E_ = 0.270 and *F*_ST_ = 0.006) were comparable to those obtained with other genetic markers. Of all genes, 5.5% were identified as outliers with *F*_ST_ at the 95% confidence level, while 14% were identified as candidates for local adaptation with the Bayesian method. There was some overlap between the two gene sets. More than half of the candidate genes for local adaptation were specific to the warmest population, about 20% to the most arid population, and 15% to the coldest and most humid higher altitude population. These adaptive trends were consistent with the genes’ putative functions and the divergence in quantitative traits noted among the populations. The results suggest that an approach separating the locus and population effects is useful to identify genes potentially under selection. These candidates are worth exploring in more details at the physiological and ecological levels.

## Introduction

Identifying genomic regions involved in local adaptation is a challenging task for plant evolutionary biologists, breeders, and conservationists. It is particularly challenging in forestry because trees such as conifers are among organisms with largest genome size (Wakamiya *et al*. 1993; Murray 1998) and a rapid decay of linkage disequilibrium (LD), most often within gene limits (Neale & Savolainen 2004). Such factors are likely to reduce the efficiency of genome scans based on intergenic or anonymous markers in detecting adaptive variation linked to coding regions. They also make it hard to adequately disentangle the effects of historical or demographic factors from those of natural selection since a higher marker density would be needed to attain this goal (Storz 2005; Wright & Gaut 2005). Indeed, evidence for discrepancies in population differentiation estimates due to the limited number of loci and their sparse distribution on the genome has been reported (Nybom 2004).

Until recently, most adaptive genome scans in plants have relied on the identification of quantitative trait loci (QTL) by using mapping approaches for inbred or outbred pedigrees (e.g. Frewen *et al*. 2000; Hurme *et al*. 2000; Bradshaw & Schemske 2003). They also relied extensively on DNA markers from noncoding or anonymous regions of the genome such as random amplified polymorphic DNA (RAPD), amplified fragment length polymorphism (AFLP), or simple sequence repeats, also known as microsatellites (SSR). Despite its great utility, the QTL approach is difficult to apply in cases where traits cannot be measured before many years of testing in common gardens, or when test crosses cannot be experimentally performed (Storz 2005). It also steps short from elucidating the genetic basis of complex traits when several genes and environmental factors regulate the phenotype under study (e.g. see Howe *et al*. 2003; Erickson *et al*. 2004) or when QTLs encompass large genomic regions.

An alternative approach consists in using a multilocus genome scan that identifies outlier loci for population differentiation by screening genomes for genetic polymorphisms directly into natural populations (Luikart *et al*. 2003; Schlötterer 2003; Storz *et al*. 2004). This approach has the advantage of not requiring knowledge of genetic divergence in quantitative traits between populations. It also does not require a complete knowledge of the DNA sequence underlying the loci investigated (Storz 2005). Its main assumption is that when an adaptive mutation is driven to fixation by selection at a specific locus, this fixation will lead to a joint fixation of the neutral linked variants (Maynard Smith & Haigh 1974; Kaplan *et al*. 1989; Stephan *et al*. 1992), which can be maintained in small fractions in the species genome even when recombination randomizes associations between the selected site and the linked neutral variants (Storz 2005). This process is known as genetic hitch-hiking and can be easily detected since the selected locus and its linked regions exhibit a variation pattern that is distinct from the rest of the genome, mainly a skewed allele frequency distribution, a lower within-population variation, and a higher differentiation for the locally adapted populations (Vasemägi & Primmer 2005).

To date, genome scans for the identification of loci or genes involved in local adaptation have been extensively used in humans (e.g. Altshuler *et al*. 2000; Akey *et al*. 2002; Kayser *et al*. 2003) and for some model organisms (e.g. Cork & Purugganan 2005; Wright *et al*. 2005), but they are still in their infancy for most species including conifers. In most cases, because of the anonymous or intergenic nature of the markers used, these scans were limited to the identification of outlier loci without a special focus on identifying the underlying genes or possible causes at the molecular level (Scotti-Saintagne *et al*. 2004; Krutovsky & Neale 2005; Bousquet *et al*. 2007; Tsumura *et al*. 2007). In taxa characterized by large genome sizes and low LD such as conifers, these approaches might effectively miss regions of the genome involved in adaptation because coding genes that can be the target of selection are embedded in large recombining noncoding regions. Such a genome configuration necessitates high marker densities to detect the effect of natural selection on linked genomic regions. This configuration becomes even more problematic in species characterized by large genomes. In addition, the accuracy of anonymous or intergenic markers such as RAPDs, AFLPs, and SSRs in estimating allelic variation within or between populations (Hedrick 1999; Isabel *et al*. 1999; Mariette *et al*. 2002) and consequently, in detecting natural selection along a species genome, might be decreased by partial DNA digestion or high mutation rates, two potential sources of homoplasious variation.

One way to circumvent these limitations is to rely on polymorphisms located in genes or gene regions closely linked to them. With sequencing becoming more affordable, large-scale sequencing of expressed sequence tags (ESTs) may represent a useful starting point for mining DNA polymorphisms located directly into genes for such species. The detection of DNA polymorphisms in large numbers of expressed genes and their use in population studies has been shown to be feasible, using EST-linked microsatellites (e.g. Vasemägi *et al*. 2005; Oetjen & Reusch 2007) and single nucleotide polymorphisms (SNP, e.g. Kimura *et al*. 2007; Zayed & Whitfield 2008).

Recently, Pavy *et al*. (2006) identified more than 12 000 SNPs from the clustering of about 50 000 ESTs from the white spruce [*Picea glauca* (Moench) Voss] genome (Pavy *et al*. 2005). The discovery of such a large number of SNPs provides a useful starting point to perform a genome-wide SNP scan in natural populations of a conifer species in order to identify candidate genes underlying adaptation (Bouck & Vision 2007; Bousquet *et al*. 2007). Although the characterization of variation in quantitative characters is not an essential condition for the success of such a scan (Storz 2005), one potentially powerful approach would be to link this scan to populations previously characterized for adaptive quantitative characters in common garden studies. In doing so, population sampling could be orientated more effectively based on knowledge of variation in quantitative characters, thus reducing the rate of false–positives. For white spruce, many large-scale replicated provenance tests have already been established for the first-generation breeding cycle and for gene conservation purposes. These tests indicate significant among-population genetic variation for quantitative traits related to growth, phenology, and wood characters (Li *et al*. 1997; Jaramillo-Correa *et al*. 2001), but corroborative evidence using a genome-wide scan has not yet been investigated.

In the present study, we took advantage of the new EST resource to assess the effectiveness of a genome-wide SNP scan focused on expressed genes in detecting adaptive polymorphism in six natural populations of white spruce. We also tried to determine whether the adaptive patterns observed at some genes could be associated, at least qualitatively, with the bioclimatic conditions or phenotypic attributes of the populations, as well as with the functional properties of the genes. To identify genes with an adaptive genetic pattern, we used two different approaches: one relying on estimates of overall among-population differentiation (Beaumont & Nichols 1996), and the other based on a Bayesian method aimed at identifying local adaptation (Beaumont & Balding 2004). The latter has the advantage of separating the locus and population effects.

## Materials and methods

### Gene selection

A total of 656 genes were chosen and resequenced among a sample of 16 500 annotated unigenes derived from the assembly of around 50 000 white spruce ESTs (Pavy *et al*. 2005). The ESTs were from 5′ and 3′ reads and were generated from 16 cDNA banks. The average length of high-quality reads was 645 nucleotides (Pavy *et al*. 2005). The present subset of 656 genes represents candidates for regulatory function, wood formation, plant growth, or phenology. Various publicly accessible data banks were used to retrieve annotations and identify functional attributes of the genes including spruceDB (http://biodata.cbri.umn.edu/spruce/), ForestTreeDB (http://foresttree.org:8680/DB/nimbus/project.do), PFAM (http://www.sanger.ac.uk/Software/Pfam/), and *Arabidopsis* databases (http://arabidopsis.med.ohio-state.edu/AtTFDB/andhttp://datf.cbi.pku.edu.cn*/*). Because of EST redundancy, high probability *in silico* SNPs were identified for many of these genes (Pavy *et al*. 2006). Resequencing was conducted to confirm these SNPs and discover new ones.

### SNP discovery

The 656 candidate genes were resequenced for the two white spruce parents of a linkage mapping population in order to identify SNPs enabling gene mapping in a large F_1_ population (Pavy *et al*. 2008). The same set of SNPs was used in the present survey. Primers for amplification and resequencing were generally placed in 5′ or 3′ untranslated regions of the genes to increase specificity. Methods for the identification of coding regions and primer design for amplification and resequencing are reported elsewehere (Pavy *et al*. 2008). Each gene was also resequenced from DNA of a megagametophyte, the tissue surrounding the embryo of a conifer seed, in order to identify and exclude paralogous SNPs (Pavy *et al*. 2008). Because of the haploid nature of conifer megagametophytes, no polymorphism is expected in haploid DNA sequences. Any exception to the rule indicates polymorphism of paralogous nature that was not considered further. A final screening was conducted to eliminate SNPs with variable flanking regions (e.g. with highly repetitive sequences, palindromes, polymorphism located too close of each other) in order to eliminate SNPs with low probability of genotyping success using the GoldenGate assay (Fan *et al*. 2003; see below). Of the 656 candidate genes, 487 could be successfully amplified. A subset of 424 genes containing a total of 768 orthologous SNPs were used for the construction of the SNP array.

### Population sampling and DNA isolation for the population scan

**Fig. 1:**
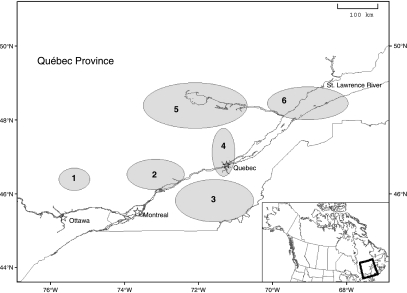
Geographical distribution of the six populations (ecoregions) analysed.

Trees were sampled from natural populations of white spruce distributed in different ecological regions in Québec. They extended from the temperate hardwood to the boreal conifer forest (Fig. 1). In total, six broad populations representative of as many distinct ecological regions were analysed in the present study. Previous studies showed that these six populations were in drift–migration equilibrium, exhibited no significant genetic differentiation at neutral loci, but were significantly differentiated in quantitative traits related to wood density, phenology, and growth, as determined in common garden studies and by *Q*_ST_ differentiation estimates (Jaramillo-Correa *et al*. 2001). Trees were represented by ramets maintained in a clonal bank and open-pollinated families grown in common garden tests previously established in 1979 and 1980 (Li *et al*. 1993, 1997). Bioclimatic data and phenotypic attributes of these populations are presented in Table 1. From 20 to 34 trees per population could be sampled, for a total of 158 trees. For each tree, DNA was extracted from dormant buds using a DNeasy Plant mini kit according to the manufacturer's instructions (QIAGEN).

**Table 1:** Average climatic parameters and quantitative traits for the six sampled populations (ecoregions) of white spruce*

	Population					
	
Climatic parameters and traits	No. 1	No. 2	No. 3	No. 4	No. 5	No. 6
Elevation (metres)	269	211	205	771	334	198
Annual number of degree-days†‡	1570	1633	1789	970	1305	1353
Precipitation from June to August (millimetres)†	305	319	348	430	345	292
Aridity index†	4.1	4.0	3.8	0.9	2.5	4.2
Total annual precipitation (millimetres)†	1058	1111	1127	1599	1090	1079
Annual number of days without frost†	176	181	194	139	161	171
Annual average temperature (°C)†	3.3	3.6	4.8	–0.3	1.1	2.2
Maximum temperature (°C)†	33.0	32.8	32.7	29.2	31.7	31.6
Minimum temperature (°C)†	–36.8	–34.9	–32.1	–37.7	–38.4	–33.4
Height (centimetres)§	516 (4)	494 (4)	447 (4)	466 (5)	493 (3)	449 (5)
Diameter at breast height (centimetres)§	7.1 (0.1)	6.9 (0.1)	5.9 (0.1)	6.5 (0.1)	6.7 (0.1)	6.0 (0.1)
Wood density (g/cm^3^)§	0.361 (0.002)	0.369 (0.002)	0.382 (0.002)	0.368 (0.003)	0.367 (0.002)	0.387 (0.001)
Date of budset (julian days)§¶	230 (2)	236 (1)	236 (1)	229 (1)	231 (1)	235 (1)
Date of budburst (julian days)§¶	143 (< 1)	143 (< 1)	143 (< 1)	144 (< 1)	143 (< 1)	143 (< 1)
Duration of growing season (days)§	87 (2)	93 (1)	94 (1)	84 (1)	87 (1)	92 (1)

* Traits were measured in a common garden study (see Materials and Methods);

† average estimates are for the reference period 1971–2000 and were obtained by using the biosim climate simulator (Régnière 1996);

‡ the number of degree-days corresponds to the cumulative number of degrees above 5 °C;

§ quantitative traits were measured at age 22 on half-sib progenies raised in a common garden in three replicated sites for height, diameter at breast height, and wood density, and at age 3 at a nursery site with replications for phenological traits; numbers in parentheses indicate the standard errors;

¶ the number of julian days corresponds to the number of days elapsed since noon Greenwich Mean Time on January 1st.

### SNP genotyping

SNP genotyping of the 158 sampled individuals was performed by using the Illumina SNP bead array platform (Illumina, San Francisco, California) and the GoldenGate allele-specific extension assay, a highly multiplexed genotyping assay (Fan *et al*. 2003; Shen *et al*. 2005). It was carried in 96-well plates using 2 µg of DNA extract normalized at 50 ng/µL for each sample. Briefly, the GoldenGate assay consists in genotyping genomic DNA directly without the need for polymerase chain reaction (PCR) amplification by hybridizing two allele-specific (ASO) and one locus-specific oligos (LSO) with each DNA sample in the array matrix. It allows highly multiplex genotyping, up to 1536 SNPs in the GoldenGate genotyping assay. Further details about this technique can be found in Shen *et al*. (2005).

GenCall and GenTrain scores were used to evaluate the accuracy and efficiency of SNP genotyping. These scores reflect the degree of separation between homozygote and heterozygote clusters for each SNP (Fan *et al*. 2003). The lowest acceptable score was set at 0.25, which represents a stringent criterion used in human genetic studies (http://www.illumina.com/; Fan *et al*. 2003). In the present study, this threshold corresponded to SNPs with accurate scoring for at least 95% of the individuals, with most successful SNPs scored for over 99% of the individuals analysed.

### Data analysis

Genetic diversity was estimated for each population by calculating the percentage of polymorphic SNPs (*P*_O_), the mean number of alleles per SNP (*A*), observed (*H*_O_) and expected (*H*_E_) heterozygosities (unbiased, Nei 1978), and the within-population fixation index (*F*). The deviation of fixation indices from zero was tested by 10 000 permutations of alleles between individuals. Population departure from Hardy–Weinberg equilibrium was also tested. Among-population differentiation estimates (*F*_ST_) were calculated and their deviation from zero was tested by 10 000 allele permutations. These various parameters were calculated with arlequin version 2.3 software (http://anthro.unige.ch/arlequin). Detection of genes carrying the signature of natural selection or ‘outliers’ was first performed with the fdist2 program that uses the summary-statistic approach described in Beaumont & Nichols (1996) and further developed in Beaumont & Balding (2004). This program is available at http://www.rubic.reading.ac.uk/~mab/software/fdist2.zip. It first calculates *F*_ST_ for each sampled locus with the Weir & Cockerham (1984) formula (#10, page 1364). It then uses coalescent simulations to generate a null distribution of *F*_ST_values based on an infinite island model for the populations and an infinite allele model for polymorphism (Beaumont & Nichols 1996). Loci with an unusually high or low *F*_ST_ value conditional on heterozygosity are considered as potentially under selection. In this study, we simulated the neutral distribution of *F*_ST_ with 60 000 iterations at the 95% confidence level.

The program newfst developed by Beaumont & Balding (2004) was also used to identify genes subject to selection. This program relies on a Bayesian model to generate *F*_ST_ values by implementing a Metropolis Hastings Markov chain Monte Carlo (MCMC) algorithm based on the likelihood of allele counts. It has the advantage of disentangling the locus effect (α_*i*_), the population effect (β_*j*_), and the interaction between the locus and the population effects (γ_*ij*_). In general, a large positive α_*i*_ indicates the presence of a positive selection on the studied gene, while a large positive γ_*ij*_ indicates an important locus–population interaction, thus a potentially advantageous mutation that would be locally adapted to a particular population (Beaumont & Balding 2004). The probability densities for *F*_ST_ values were obtained with the assumption of independent, lognormal (1, 1.8, 0.5) prior distributions for the α_*i*_, β_*i*_, and γ_*ij*_. For this, Gaussian kernel density estimation was used based on 160 000 iterations of the Metropolis algorithm with a thinning interval of 320. The convergence of the 10 000 parameters series α_*i*_, β_*i*_, and γ_*ij*_ generated by the MCMC algorithm was tested with the Heidelberger & Welch (1981) convergence diagnostic using the coda package of r version 2.2.1. r is an open-source statistical software available at http://www.r-project.org/. In a first step, we set the confidence level at 95% to identify large positive values of α_*i*_ and γ_*ij*_. Genes identified with a positive α_*i*_ value were called outliers, while those identified with a large positive γ_*ij*_value were termed candidate for local adaptation throughout the paper. Although only two of them reached the 95% confidence level of the simulations (see Results), those with high positive γ_*ij*_values (above 0.10) were retained for further investigation as they possibly reflected true adaptive trends. To compare these results to those obtained with the summary-statistic method (fdist 2), we adjusted the confidence levels to 90% and 99% for newfst and fdist 2, respectively. This 10-fold difference in confidence level was suggested by Beaumont & Balding (2004) to make the two methods comparable for their own data by maintaining the same rate of false-positives.

In an attempt to find corroborative evidence for the genetic patterns observed, we qualitatively assessed the presence of possible relationships between the genes identified as candidate for local adaptation and the bioclimatic and phenotypic attributes of each population. The bioclimatic parameters included the annual number of degree-days, the aridity index, the total precipitation during summer, the total annual precipitation, the annual number of days without frost, the annual average temperature, and the maximum and minimum temperatures (Table 1). The quantitative traits were estimated from common garden studies (see Li *et al*. 1993, 1997 for study design) and included tree height, diameter at breast height (d.b.h.), and wood density at age 22, as well as the date of budburst, the date of budset, and the duration of the growing season at age 3. We also looked at the current functional annotation and classification of the candidate genes for local adaptation as described above. However, the present effort of linking statistical and functional inferences has to be considered as preliminary since many of the genes used in this study are still not well characterized at the functional or transcriptional level.

## Results

### Genotyping success

Among the 768 SNPs analysed, 166 failed to provide an acceptable GenTrain quality score, 68 had an acceptable quality score but were monomorphic in all samples, and the remaining 534 (70%) were polymorphic with a high quality score in more than 95% of the sampled individuals (Table 2). The 534 successful SNPs were located on 345 expressed genes including 260 (75%) that were regulatory. Regulatory genes were identified by comparing spruce sequences to *Arabidopsis* transcription factors (http://arabidopsis.med.ohio-state.edu/AtTFDB/ and http://datf.cbi.pku.edu.cn/) and to PFAM domains (http://www.sanger.ac.uk/Software/Pfam/) (Pavy *et al*. 2005). Given that the complete sequence of the *Picea* genome is not available to fully validate SNPs before spotting them on the SNP-bead array, the proportion of SNPs successfully genotyped in the populations (70%) compares well with that for human SNPs using the same technology (80%) (Shen *et al*. 2005). The 534 successful SNPs and their 345 genes were distributed along the 12 linkage groups of white spruce (Pelgas *et al*. 2006) at a rate of 19–31 gene locus per linkage group (Pavy *et al*. 2008).

**Table 2:** Genotyping success of SNP markers and genes using the Illumina GoldenGate multiplex assay

Category	Number of SNPs	Percentage of SNPs	Number of genes	Percentage of genes
Failed SNPs (Gentrain score < 0.25)*	166	21.6	20	4.7
Successful SNPs but monomorphic	68	8.9	27	6.4
Successful SNPs and polymorphic	534	69.5	345	81.4
Total	768	100	424	100

* The GenTrain score reflects the degree of separation between homozygote and heterozygote clusters for each SNP and the placement of the individual call within each cluster (Shen *et al*. 2005).

### Genetic diversity

**Fig. 2:**
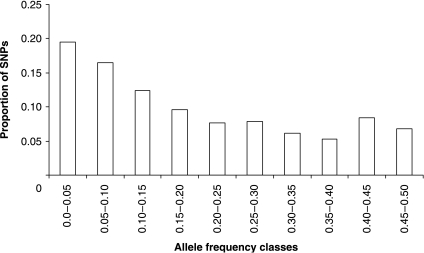
Allele frequency distribution at 534 SNP loci.

No more than two nucleotides could be detected per SNP. Accordingly, the mean number of alleles per polymorphic locus (*A*) per population ranged from 1.90 to 1.94 with a grand mean of 1.92. About 65% of all SNPs had an overall frequency equal to or larger than 0.10 (Fig. 2) and were considered common SNPs. Out of 3204 tests performed, only two departed from Hardy–Weinberg equilibrium (at α = 0.05). Overall genetic diversity (i.e. considering all polymorphic SNPs together) expressed by the average observed heterozygosity (*H*_O_) ranged from 0.263 to 0.293 among populations with a grand mean of 0.276 (SD = ±0.011). Average unbiased expected heterozygosity (*H*_E_) was generally slightly lower than *H*_O_ and ranged from 0.266 to 0.274 per population with a grand mean of 0.270 (SD = ±0.003). The average within-population fixation index *F (*averaged over all loci in each population) showed a significant excess of heterozygotes in population nos 1, 2, and 4 (Table 3). The largest number of SNPs with a significant excess of homozygotes was in population nos 3 (14 SNPs for 14 genes) and 6 (15 SNPs for 14 genes).

**Table 3:** Genetic parameters of the six sampled populations (ecoregions) of white spruce*

Population	Number of trees sampled	A	*H*_O_	*H*_E_	*F*
No. 1	25	1.93	0.293	0.274	–0.073†
No. 2	31	1.94	0.277	0.273	–0.019†
No. 3	25	1.91	0.263	0.266	0.011
No. 4	20	1.90	0.283	0.268	–0.059†
No. 5	34	1.93	0.269	0.269	–0.001
No. 6	23	1.92	0.271	0.269	–0.008
Mean	26.3	1.92	0.276	0.270	–0.025
SD	5.2	0.01	0.011	0.003	0.033

* *A* is the mean number of alleles per SNP*, H*_O_is the average observed heterozygosity; *H*_E_is the average unbiased expected heterozygosity (Nei 1978); *F* is the average fixation index; SD is the standard deviation;

† *P* ≤ 0.01 based on 10 000 permutations between individuals within the same population.

### Genetic differentiation among populations and detection of genes targeted by selection

**Fig. 3:**
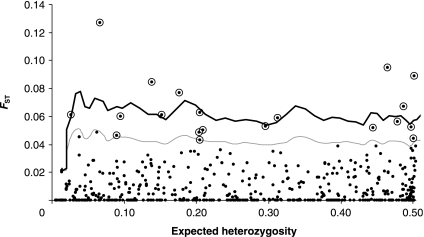
Outlier detection and distribution of empirical *F*_ST_ values as a function of expected heterozygosity. The solid line indicates the 99% upper and lower confidence levels and the grey line indicates the 95% upper and lower confidence levels, as estimated using the summary-statistic method of Beaumont & Nichols (1996). The identification of 20 outlier SNPs (above the 95% confidence level, fdist 2) is indicated by circled dots.

**Fig. 4:**
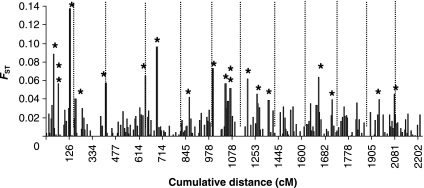
Distribution of empirical *F*_ST_ values over the 12 linkage groups of white spruce with linkage groups (LG) order following Pelgas *et al*. (2006). The vertical dotted lines indicate approximate boundaries between consecutive linkage groups. The identification of 20 outlier SNPs (above the 95% confidence level, fdist 2, Fig. 3) is indicated by asterisks above vertical solid lines. cM is centimorgans.

**Fig. 5:**
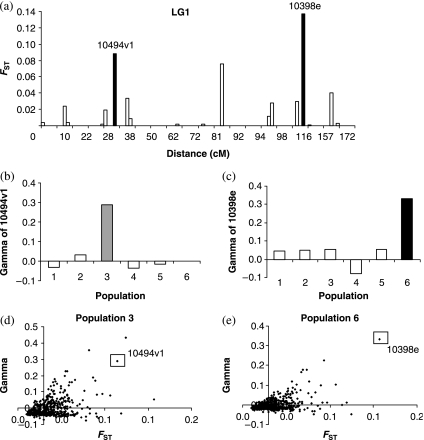
(a) Distribution of empirical *F*_ST_ values over linkage group 1 (LG1) with focus on two outlier SNPs (above the 99% confidence level, fdist 2) in grey and black. cM is centimorgans. (b) and (c) Distribution of the mean gamma values (γ_*ij*_) of the two outlier SNPs highlighted in (a) and indicative of local adaptation among the six populations studied. Gamma values were estimated with a Bayesian method and reflect the interaction between the locus and the population effects. (d) and (e) Distribution of gamma values (γ_*ij*_) for all SNPs in populations no. 3 and no. 6 relative to their overall *F*_ST_ values estimated with the summary-statistic method (fdist 2).

The average level of genetic differentiation among populations was extremely low (*F*_ST_ = 0.006) but significantly different from zero at the 95% confidence level. *F*_ST_ values per SNP ranged from –0.019 to 0.137, with 73 SNPs (14%) having a value significantly different from zero at the 95% confidence level. In this study, negative *F*_ST_ values were considered equal to zero because they do not bear any biological interpretation. In general, *F*_ST_ values followed an L-shaped distribution with 223 SNPs (42%) having an *F*_ST_ value below or equal to zero, and 21 SNPs (4%) in the tail of the distribution with a value higher than 0.04.

fdist 2, which is based on the summary-statistic method, identified 20 SNPs (3.7%) located on 19 genes (5.5%) with an *F*_ST_value above the 95% confidence level. Most of the outlier SNPs had an empirical *F*_ST_ higher than 0.04 (Fig. 3) and were distributed over the 12 linkage groups (Fig. 4). As expected, newfst, which is based on a Bayesian method that isolates the locus and population effects, provided a much lower figure at the same 95% confidence level: only two SNPs (0.4%) located on two different genes (0.6%) located on two different linkage groups carried the signature of positive selection when the locus effect parameter (α_*i*_) alone was considered, while none carried the signature of local adaptation when considering the locus × population parameter (γ_*ij*_). However, 49 SNPs (9.2%) located on 47 genes (13.6% including the two with a high positive α_*i*_ value) exhibited a relatively large positive mean γ_*ij*_value (> 0.10) in one or two populations while maintaining negative or close to zero mean γ_*ij*_values in the other populations (Fig. 5b–e). This can be indicative of a possible adaptive trend in some populations. These SNPs also had a significant average *F*_ST_after permutations (Fig. 5a) and were therefore considered as candidate for local adaptation. Conversely, no SNP had a significantly negative α_*i*_value, indicating the absence of balancing selection. Most outliers identified with fdist 2 were also identified as candidate for local adaptation by newfst. These 49 SNPs were distributed over the 12 linkage groups of white spruce and included 21 SNPs (20 genes) located on exons and 28 SNPs (27 genes) on introns or the 5′UTR and 3′UTR regions (χ^2^ = 0.02, d.f. = 1, *P* = 0.88). Those located on exons included 10 synonymous and 11 nonsynonymous SNPs (χ^2^ = 0.99, d.f. = 1, *P* = 0.32), and the number of those with low allele frequency classes (below 10%) was not significantly different from the number of those considered as common (χ^2^ = 0.12, d.f. = 1, *P* = 0.73). When adjusting the confidence levels to 99% and 90% for fdist 2 and newfst, respectively (Beaumont & Balding 2004), the results became much similar: nine SNPs (1.7%) located on eight different genes (2.3%) could be declared outliers with fdist 2, compared to five SNPs (0.9%) located on five genes (1.4%) with newfst.

### Geographical and functional trends

The number of candidate SNPs for local adaptation was significantly different among the six populations (χ^2^ = 39.10, d.f. = 5, *P* < 0.01), with the largest number being in population no. 3, then population nos 6 and 4 (Table 4). These three populations harboured 84% and 88% of the outlier SNPs identifed by fdist 2 at the 95% and 99% confidence levels, respectively. Population no. 3 that harboured the largest number of candidate SNPs for local adaptation had the warmest environmental conditions with the annual average temperature, number of degree-days and days without frost at the high end of the spectrum observed among the six populations (Table 1). Interestingly, growth parameters were at the low end. Population no. 6 that came second in terms of the number of candidate SNPs for local adaptation was on the high end of the aridity spectrum and its trees had the highest wood density (Table 1). Population no. 4 with the third largest number of candidate SNPs for local adaptation was the most elevated in altitude. It was also the most humid and the coldest with an annual average temperature of –0.3 °C (Table 1). Its trees had the shortest duration of growing season with the latest budburst and earliest budset (Table 1).

**Table 4:** List and properties of the 49 candidate SNPs for local adaptation representative of 47 genes*

SNP†	Locus‡	Gene family	tblastx (e-value)	*H*_E_§	*F*_ST_ (fdist 2)¶	Population with mean γ_*ij*_ > 0.10**	Putative biological function††
01424a	AT1G50640.1	AP2	3E-22	0.090	0.009	No. 3	GR, RE, WF, BS, AS
01471a	AT3G04730.1	Auxin	6E-28	0.480	0.004	No. 3	GR, RE, WF
01687n	AT1G62360.1	Homeobox	7E-68	0.446	0.067	No. 4	GR
02603e	AT1G25280.2	Tubby	3E-46	0.415	0.028	No. 5	GR
02892v3	AT3G23920.1	Beta amylase	3E-90	0.088	0.060	No. 5	GR, AS
03056e	AT3G12130.1	C3H RING finger	3E-60	0.413	0.095	No. 3	GR, RE
03579f	AT1G76880.1	Trihelix	3E-46	0.470	0.044	No. 4	PH, RE
03579 g	AT AT1G76880.1	Trihelix	3E-46	0.475	0.0401	No. 4	PH, RE
03693f	AT2G37630.1	Myb	2E-89	0.333	0.009	No. 6	GR, WF, AS
03713 m	AT1G66230.1	Myb	4E-72	0.426	0.007	No. 3	GR, WF, AS
04148e	AT1G69780.1	Homeobox	5E-33	0.482	0.015	No. 1 and No. 3	GR
04190t1	AT5G02810.1	C2C2-colike	6E-23	0.012	0	No. 1	PH, RE
04514 g	AT3G20770.1	Pseudouridine synthase A	2E-22	0.190	0.048	No. 4	GR, RE, AS
04885a	AT1G50640.1	AP2	3E-26	0.105	0.005	No. 3	GR, RE, WF, BS, AS
05090v1	AT2G19940.1	Gamma-glutamyl- phosphate reductase	1E-136	0.462	0.052	No. 4	AS
05937v1	AT5G49580.1	Chaperone protein DnaJ	4E-24	0.126	0.032	No. 3	AS
05953 g	AT5G17600.1	C3H RING finger	3E-47	0.188	0.063	No. 6	GR, RE, AS
06620 g	AT4G04885.1	C2H2 zinc finger	1E-24	0.414	0.032	No. 6	GR, AS
06684v1	AT1G70320.1	E3 ubiquitin protein ligase	4E-56	0.058	0.048	No. 1	GR, RE, AS
07106p1	AT1G04140.2	WD40	4E-85	0.289	0.059	No. 3	GR, RE, PH
07248e	AT3G16830.1	WD40	1E-142	0.448	0.089	No. 4	GR, RE, PH
07393f	AT1G69440.1	Argonaute	7E-56	0.195	0.050	No. 1 and No. 5	GR
07506a	AT1G60710.1	Oxidoreductase	1E-152	0.482	0	No. 3	GR, BS
07604v4	AT5G26680.1	Flap endonuclease-1	1E-41	0.192	0.043	No. 6	GR, WF
07977f	AT2G47900.2	Tubby	1E-82	0.367	0.003	No. 3	GR
08080 m	AT4G38620.1	Myb	1E-81	0.084	0.046	No. 3 and No. 6	GR, WF, AS
08177v1	AT4G11790.1	Ran binding protein	2E-44	0.403	0.016	No. 3	GR
08349a	AT1G25280.2	Tubby	9E-76	0.264	0.015	No. 3	GR
08438b	AT3G55770.2	LIM	4E-84	0.328	0	No. 2 and No. 6	WF
08987p1	AT1G22190.1	AP2	1E-26	0.130	0.029	No. 5	GR, RE, WF, BS, AS
09562a	AT2G19810.1	C3H RING finger	6E-83	0.109	0.011	No. 3	GR, RE, AS
09644v2	AT1G60420.1	Peroxidase	6E-71	0.326	0.024	No. 3	WF, AS
09863a	AT2G01570.1	GRAS	1E-140	0.231	0.011	No. 3	GR, WF
09889b	AT3G12390.1	NAC	6E-48	0.241	0.025	No. 3	GR, BS
09982e	AT1G10200.1	LIM	1E-73	0.177	0	No. 5	WF
10016v1	AT5G06720.1	Peroxidase	7E-91	0.443	0.056	No. 3	WF, AS
10125n	AT3G15610.1	WD40	1E-128	0.469	0.037	No. 6	GR, RE, PH
10398e	AT1G08830.2	Superoxide dismutase	3E-49	0.056	0.127	No. 6	PH, AS, BS
10494v1	AT1G55670.1	Photosystem I reaction centre subunit V	6E-53	0.124	0.084	No. 3	PH
10583v1	AT2G21490.1	Dehydrin	1E-21	0.473	0.035	No. 4	AS
10614t2	PTU09554	AGP	3E-85	0.338	0.013	No. 3	WF
11176a	AT4G24020.1	Nin-like	2E-58	0.141	0.061	No. 4	BS
11176b	AT4G24020.1	Nin-like	2E-58	0.160	0.077	No. 1 and No. 6	BS
12347a	AT1G16070.2	Tubby	2E-31	0.105	0.008	No. 3	GR
13634e	AT3G13040.2	Myb	7E-34	0.486	0.006	No. 3	GR, WF, AS, BS
14328e	AT4G32600.1	C3H RING finger	4E-76	0.143	0.023	No. 1	GR, RE, AS
14745f	AT1G78070.2	WD40	5E-70	0.394	0.028	No. 3	GR, RE, PH
15115e	AT5G47390.1	Myb	4E-58	0.413	0.052	No. 3	GR, WF, AS, BS
90002e	AT5G47670.2	CCAAT box-binding factor E22	1E-54	0.220	0.036	No. 3	RE, PH

* Candidate SNPs for local adaptation are those exhibiting a mean locus–population interaction parameter (γ_*ij*_) higher than 0.10 based on the Bayesian method;

† SNP annotations and nomenclature are detailed in Pavy *et al*. (2008); related transcript sequences can be found in the spruce gene database at http://biodata.cbri.umn.edu/spruce/;

‡ Sequences were paired to an *Arabidopsis* locus based on sequence highest homology through tblastx searches against *Arabidopsis* blastsets database (ftp://ftp.arabidopsis.org/home/tair/Sequences/blast_datasets/TAIR7_blastsets/TAIR7_seq_20070320);

§ Expected heterozygosity;

¶ *F*_ST_ was calculated as an estimate of Weir & Cockerham's (1984) β statistics as described in Beaumont & Nichols (1996);

** The γ_*ij*_ parameter is the mean locus–population interaction parameter as defined by Beaumont & Balding (2004);

†† PH, phenology; GR, growth; RE, reproduction; AS, abiotic stress; BS, biotic stress; WF, wood formation.

In terms of gene ontology based on biological processes and in concordance with the physiological patterns described above, population no. 3 was, among the six populations surveyed, the one with the largest number of candidate adaptive genes involved in growth (19 genes) followed by abiotic stress (10 genes), reproduction, and wood formation (nine genes each). In population no. 6 characterized by the most arid conditions, candidate adaptive genes affecting wood formation were the second most abundant after growth related ones. This pattern was not seen for the other populations. For population no. 4 characterized by a lower annual average temperature and a shorter growing season, a disproportionate number of candidate genes related to phenology, reproduction, and stress were observed, compared to the other populations (Table 4). Interestingly, most of the candidate SNPs for local adaptation in population nos 4 and 6 were located in introns or in 5′UTR or 3′UTR regions, and half of them were located in exonic regions in population no. 3, almost equally divided between synonymous and nonsynonymous SNPs. No significant difference could be observed in the number of candidates for local adaptation between regulatory and nonregulatory gene categories (χ^2^ = 0.55, d.f. = 1, *P* = 0.46). The gene family with the largest number of candidate genes for local adaptation was that for the *Myb* genes, which play an important role in plant growth and wood and lignin formation (Bedon *et al*. 2007). However, this family was over-represented in the gene sampling, which makes this trend only indicative.

## Discussion

### Genome scans in conifer species

To the best of our knowledge, this is the first report on a genome-wide SNP scan of expressed genes in a nonmodel species. It is also the first one to be conducted in natural conifer populations for which significant genetic differentiation in quantitative traits has been demonstrated from common garden studies (Jaramillo-Correa *et al*. 2001). The average among-population genetic differentiation estimated by *F*_ST_ was very low (0.006 or 0.60%), but comparable to that previously reported with allozymes and expressed sequence tag polymorphisms (ESTP) (mostly indels) in the same populations (*G*_ST_ = 1.4% and 2.0%, respectively; Jaramillo-Correa *et al*. 2001). It was also comparable to that observed in other white spruce populations using nuclear molecular markers (*G*_ST_ = 1.6%; Godt *et al*. 2001) and in black spruce populations in the same region (Isabel *et al*. 1995; Perry & Bousquet 2001), a species reproductively well isolated from white spruce but similar in terms of demography and population genetic parameters. As expected, white spruce harboured little population structure at the nuclear DNA level in the region surveyed.

When compared with other SNP studies in expressed gene regions, the average observed heterozygosity (*H*_O_ = 0.276) fell in the range of that observed in other species such as humans (Moffatt *et al*. 2000; Beaty *et al*. 2005), maize (Ching *et al*. 2002), and *Arabidopsis* (Ostrowski *et al*. 2006). However, direct comparisons of heterozygosity estimates to those obtained with other markers in conifers (allozymes, RAPDs, AFLPs, SSRs, ESTPs) can be misleading. This is because SNPs are diallelic and have relatively low mutation rates (10^−8^–10^−9^; Brumfield *et al*. 2003). Also, plant coding regions generally have lower nucleotide diversity than noncoding or untranslated regions (e.g. Zhu *et al*. 2003; Guillet-Claude *et al*. 2004), which may further bias the comparison with heterozygosity estimates derived from anonymous markers or DNA polymorphisms in nongenic regions.

### Detection of candidate SNPs for adaptation

At the 95% confidence level, the observed proportion of outlier gene loci (5.5%) was slightly higher than most rates reported with AFLPs such as the 2.6% to 3.3% in Norway spruce (Acheré*et al*. 2005), the 1.4% and 3.2% in lake whitefish ecotypes (Campbell & Bernatchez 2004), and the 1.3% to 3.6% in common frog (Bonin *et al*. 2006). It was also slightly higher than the 3.0% (Akey *et al*. 2002), 5.5% (Wang *et al*. 2005), and 2.6% (Kelley *et al*. 2006) reported with SNPs from coding and noncoding regions in the human genome. However, it fell well below the 9.5% outlier rate reported in salmon populations by using EST-linked microsatellites and relying on the union of results from three different statistical approaches (Vasemägi *et al*. 2005). Apparently, the proportion of outlier genes recorded in the present study could only be temptatively compared to other studies that used summary-statistic methods. This is because all our markers were located within expressed gene regions, while most outlier studies from the literature were based on anonymous markers or markers falling outside genic regions. Moreover, caution needs to be exercised in such comparisons as the results may also depend on the statistical procedure and associated parameters used to detect outliers. For instance, our estimated proportions of outliers became lower when we adjusted the confidence levels of fdist 2 and newfst to maintain the same rate of false-positives with both programs. We expect a similar pattern in the previously published studies.

With the Bayesian method, we did not detect strong local adaptation (no positive γ_*ij*_ at the 95% or the 99% confidence levels), but 49 SNPs showed a trend towards local adaptation (γ_*ij*_value > 0.10). This is not surprising because strong local adaptive responses are more likely to be detected with this method when selective forces are important (Beaumont & Balding 2004). In the present case, the study covers only a small part of the natural range of white spruce, which extends from Alaska to Newfoundland. Also, no clear-cut bioclimatic or biogeographical differences exist between the six populations surveyed (with the exception of population no. 4 located at a higher altitude in the Laurentians), which could have facilitated gene flow among them and thus limited the efficiency of natural selection in promoting strong adaptive responses (Jaramillo-Correa *et al*. 2001). In support of this observation, a significant differentiation in adaptive traits was detected among the populations surveyed, mainly for phenological characters and wood traits, but it was moderate and *Q*_ST_ values remained below 0.30 (Jaramillo-Correa *et al*. 2001).

Another plausible explanation is that multiple gene loci may be responsible for regulating the expression of a given trait (Brem *et al*. 2002). The present gene sample, although containing many genes coding for transcription factors involved in growth, phenology, stress response and wood formation, most probably covers only a part of the gene sets controlling these traits. Even if a strong emphasis was put on sampling transcription factors in the present study (260 out of the 345 genes genotyped were of regulatory nature), the proportion of outlier SNPs was not higher for regulatory than for nonregulatory genes and only a fraction of the regulatory gene space could be tested. On the other hand, if some adaptive traits are controlled by few loci as suggested in annual plants for certain characters such as apical dominance, flowering time, or photoperiod sensitivity (Glazier *et al*. 2002), then a large number of genes needs to be surveyed to identify outliers in absence of detailed gene expression data. In such a situation, we might have missed many key adaptive genes. Lastly, the high recombination rate in conifer genomes (Brown *et al*. 2004) may have reversed the effect of selective sweeps in purging variations at linked loci (Lynch 2006). Given the long generation time and the recent Holocene history of spruce populations in eastern Canada, longer periods may in fact be needed to observe a stronger adaptive response for the genes under natural selection.

One concern related to the ability to detect outliers is the power of detection. Positive selective sweeps, usually eliciting high genetic differentiation at selected linked loci, are expected to remain for only a transient phase because recombination breaks the linkage with genes targeted by selection (Przeworski 2002). This is especially true in natural conifer populations, which harbour a rapid decay of linkage disequilibrium usually within gene limits (e.g. Brown *et al*. 2004; Neale & Savolainen 2004; Bousquet *et al*. 2007). As a result, a limited population sample size, as that used in ours and most previous studies, may reduce the efficiency of detecting low-frequency variants responsible for adaptation. Nonetheless, many association studies have shown that common alleles (*c*. 10% and more in frequency) are responsible for a large proportion of the variance in multigenic complex traits (Miller *et al*. 2000; Thumma *et al*. 2005; Broderick *et al*. 2007). In our study, the rate of candidate SNPs for adaptation belonging to the low allele frequency classes (frequency lower than 10%) was not significantly different than that found in the higher allele frequency classes (10% and more). Another possible concern with the identification of outliers is that the model assumptions and derivations used for the Bayesian simulations might not reflect the real population demography and structure. However, previous simulations demonstrated the robustness of the Bayesian model to population demography (Beaumont & Balding 2004). Moreover, the weak amount of population differentiation in white spruce because of allogamy and extensive gene flow (e.g. Jaramillo-Correa *et al*. 2001) provides little support for this concern.

The fact that a large proportion of the candidate SNPs for local adaptation were located in introns and in 5′- and 3′UTRs does not imply that these SNPs should be considered *de facto* as false-positives. Natural selection targeting intronic regions was reported in humans and chimpanzee and was shown to play an important role in species divergence (e.g. Keightley & Gaffney 2003; Drake *et al*. 2005; Gazave *et al*. 2007). Also, introns and other regions outside exons possess splicing control elements or transcriptional regulatory elements that can have multiple effects on gene expression (Majewski & Ott 2002; Carmel *et al*. 2007). Similarly, the numerous synonymous candidate SNPs for local adaptation identified in the present study should not be simply dismissed as false-positives. This is because natural selection may affect synonymous codon usage in some genes, leading to codon usage bias (Williams & Hurst 2000; Chamary & Hurst 2004). Furthermore, mutation bias or transcription related mutation/repair mechanisms may translate into unequal substitutions on each of the two DNA strands (Frank & Lobry 1999). In many cases, both mutation bias and selective pressure from functional effects on mRNA are reported to be at the origin of the asymmetry between nucleotides at a synoymous site (Frank & Lobry 1999). Conversely, genetic hitch-hiking that results from the linkage between positions along the gene can not explain these patterns. This is because linkage disequilibrium decays within a few hundreds of base pairs in gene regions in white spruce natural populations (M.-C. Namroud, C. Guillet-Claude, J. Mackay, N. Isabel & J. Bousquet, Arborea, unpublished results) as is the case in other conifers (Brown *et al*. 2004; Neale & Savolainen 2004).

### Evidence of adaptation from gene functions and patterns of population variation

The significant imbalance between populations for the number of candidate SNPs and genes for local adaptation is likely an indication of potential adaptation because an even distribution across populations would be expected with false-positives alone. Moreover, the known functions for many of these genes identified as candidate for local adaptation reflect an inclination towards maintaining biological processes that are vital for growth and survival under stressful environmental conditions. This second observation is not surprising, given the bias in the present study in selecting candidate genes for wood formation, growth, phenology, and stress response. However, only a modest proportion of candidate genes turned out to be candidates for local adaptation. In particular, one of the five genes showing the highest level of putative local adaptation belonged to the zinc finger family, which is known to control the flowering time and reproductive success (Quesada *et al*. 2005). The second one with the highest putative local adaptation belonged to the zf-B_box (superoxide dismutase) family, which plays an important role in the response to oxidative stress (Hassett & Cohen 1989), while the third one belonged to the Nin_like (ribosomal protein) family usually associated with the control of nitrogen uptake and nutrition (Schieble *et al*. 2004). These findings are in agreement with those reported by Ford (2002), who compiled more than 119 cases of plant genes or gene groups subject to positive selection in host–parasite interactions, sexual reproduction, and energy metabolism.

One interesting finding that emerges from our results and worth exploring in future physiological and ecological investigations pertains to the potential associations between the biological processes of the candidate genes for local adaptation in certain populations on one hand, and the bioclimatic and phenotypic attributes of the populations on the other hand. Although these associations are made a posteriori and are only qualitative in nature, they do provide insights about possible relationships between genetic, environmental, and quantitative trait attributes. For instance, population no. 3 had a disproportionate number of genes involved in growth in its list of candidate genes for local adaptation. At the same time, it had the warmest climate and the lowest growth (Table 1), perhaps reflecting misadaptation at the southern edge of the species natural range (Andalo *et al*. 2005). Similarly, a disproportionate number of candidate genes for local adaptation in population no. 6 were involved in wood and lignin formation, which suggests a relationship with the higher level of aridity and higher wood density observed for this population (Table 1). Higher wood density in trees is usually related to a higher proportion of latewood as an adaptation to higher aridity (Corcuera *et al*. 2004). In population no. 4 characterized by the highest altitude, coldest climate, and lowest growth potential (Table 1), there was a disproportionate representation of genes involved in phenology and stresses in the candidate genes for local adaptation, which could reflect multilocus genetic adaptation to colder climatic conditions (Table 4). Cold adaptation has been mapped on the genome of a number of forest tree species such as Douglas-fir *Pseudotsuga menziesii* (Mirb.), loblolly pine (*Pinus taeda* L.) and *Populus* spp., and it is thought to be controlled by multiple genes with small effects (Frewen *et al*. 2000; Howe *et al*. 2003). Similar QTL studies are underway for white spruce (B. Pelgas, P.G. Meirmans, C. Dhont, J. Cooke, J. Bousquet & N. Isabel, Arborea, and K. Ritland, Treenomix, personal communication).

### Study limitations and future perspectives

In this study, we used a genome-wide scan to identify genes potentially involved in local adaptation in a nonmodel undomesticated plant species. The proportion of outliers varied with the model and confidence levels used, but an approach focusing on expressed genes and taking into consideration the population effect appears promising in functional population genomic studies at the exploratory stage. This would be especially true for species similar to white spruce, with large genome sizes and a rapid decay of linkage disequilibrium in natural populations.

However, much work remains to be carried out to overcome some limitations. One of these is related to the ascertainment bias associated with the discovery of SNPs. In general, SNPs are identified in a limited discovery panel and those with common allele frequencies have more chance to be detected than rare alleles. In such cases and regardless of the ability of rare or common alleles to best account for the observed quantitative variation, rare alleles potentially involved in directional selection might be under-represented among our 534 SNPs, thus reducing the power to detect natural selection (Morin *et al*. 2004).

Another limitation pertains to the lack of information about the genes’ physiological roles. Because genotypes observed at locally adapted genes could not be directly linked to fitness-related phenotypes, genes identified as being candidate for local adaptation should be further validated and investigated using complementary approaches such as association genetic studies. Studies based on segregating unstructured populations for a number of key adaptive traits as well as QTL studies in genetically structured spruce populations are underway (N. Isabel, J. Beaulieu, J. Mackay & J. Bousquet, Arborea, personal communication). Studies are also underway to characterize patterns of variation of the candidate SNPs for local adaptation at the rangewide level across Canada. Moreover, expression profiling of these genes (J. Mackay, J. Cooke & B. Boyle, Arborea, personal communication) is expected to shed more light on the genes’ specific physiological roles, and help validate which candidate genes for local adaptation represent true positives.

New promising mass-paralleled DNA sequencing technologies (e.g. Huse *et al*. 2007) that translate into tumbling costs for sequencing and genotyping recently appeared on the market. They will likely make genome scan approaches for the search of adaptive polymorphisms more accessible than ever for nonmodel or undomesticated species. Major efforts devoted to sequencing ESTs in various species are also multiplying (e.g. Kirst *et al*. 2003; Li *et al*. 2003; Pavy *et al*. 2005; Vasemägi & Primmer 2005). With sufficient sequencing depth and appropriate statistical filtering, these EST collections might represent useful sources of common SNPs (Pavy *et al*. 2006). As a result, genome-wide SNP scans aimed at identifying outlier gene loci in population surveys should become a standard exploratory approach to detect genes under potential selection, especially for nonmodel and undomesticated species.
